# Genetic determinants of fungi-induced ROS production are associated with the risk of invasive pulmonary aspergillosis

**DOI:** 10.1016/j.redox.2022.102391

**Published:** 2022-07-04

**Authors:** Vasiliki Matzaraki, Alexandra Beno, Martin Jaeger, Mark S. Gresnigt, Nick Keur, Collins Boahen, Cristina Cunha, Samuel M. Gonçalves, Luis Leite, João F. Lacerda, António Campos, Frank L. van de Veerdonk, Leo Joosten, Mihai G. Netea, Agostinho Carvalho, Vinod Kumar

**Affiliations:** a Department of Internal Medicine and Radboud Center for Infectious Diseases, Radboud University Medical Center, Nijmegen, 6525 HP, the Netherlands; b Junior Research Group Adaptive Pathogenicity Strategies, Leibniz Institute for Natural Product Research and Infection Biology - Hans-Knoell-Institute, Jena, Germany; c Life and Health Sciences Research Institute (ICVS), School of Medicine, University of Minho, Braga, Portugal; d ICVS/3B's - PT Government Associate Laboratory, Guimarães/Braga, Portugal; e Serviço de Transplantação de Medula Óssea (STMO), Instituto Português de Oncologia do Porto, Porto, Portugal; f Serviço de Hematologia e Transplantação de Medula, Hospital de Santa Maria, Lisboa, Portugal; g Instituto de Medicina Molecular, Faculdade de Medicina da Universidade de Lisboa, Lisboa, Portugal; h Department for Genomics & Immunoregulation, Life and Medical Sciences Institute (LIMES), University of Bonn, Bonn, Germany; i University of Groningen, University Medical Center Groningen, Department of Genetics, Groningen, 9700RB, the Netherlands; j Nitte (Deemed to be University), Nitte University Centre for Science Education and Research (NUCSER), Medical Sciences Complex, Deralakatte, Mangalore, 575018, India

**Keywords:** *C. albicans*, *A. fumigatus*, Reactive oxygen species, QTLs, Stem-cell transplant recipients, Invasive aspergillosis

## Abstract

Reactive oxygen species (ROS) are an essential component of the host defense against fungal infections. However, little is known about how common genetic variation affects ROS-mediated antifungal host defense. In the present study, we investigated the genetic factors that regulate ROS production capacity in response to the two human fungal pathogens: *Candida albicans* and *Aspergillus fumigatus*. We investigated fungal-stimulated ROS production by immune cells isolated from a population-based cohort of approximately 200 healthy individuals (200FG cohort), and mapped ROS-quantitative trait loci (QTLs). We identified several genetic loci that regulate ROS levels (*P* < 9.99 × 10^−6^), with some of these loci being pathogen-specific, and others shared between the two fungi. These ROS-QTLs were investigated for their influence on the risk of invasive pulmonary aspergillosis (IPA) in a disease relevant context. We stratified hematopoietic stem-cell transplant (HSCT) recipients based on the donor's SNP genotype and tested their impact on the risk of IPA. We identified rs4685368 as a ROS-QTL locus that was significantly associated with an increased risk of IPA after controlling for patient age and sex, hematological malignancy, type of transplantation, conditioning regimen, acute graft-versus-host-disease grades III-IV, and antifungal prophylaxis. Collectively, this data provides evidence that common genetic variation can influence ROS production capacity, and, importantly, the risk of developing IPA among HSCT recipients. This evidence warrants further research for patient stratification based on the genetic profiling that would allow the identifications of patients at high-risk for an invasive fungal infection, and who would benefit the most from a preventive strategy.

## Highlights

• Several genetic loci regulate ROS levels in response to *C. albicans* and *A. fumigatus*.
• Genetic loci that regulate ROS production can be pathogen-specific, or shared between the two fungi.
• A ROS-QTL locus was significantly associated with an increased risk of developing IPA among stem-cell transplant recipients.

## Graphical abstract

**Figure:**
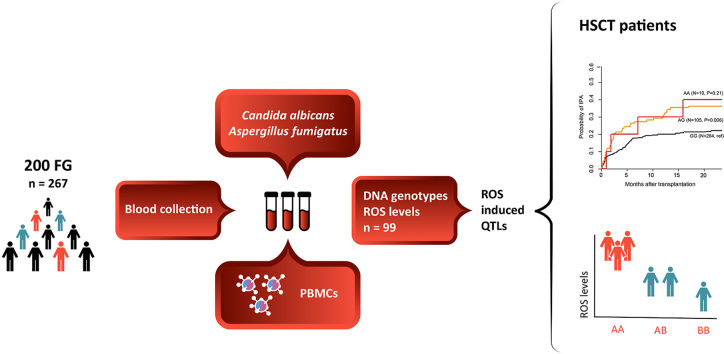
Common genetic variation can influence ROS production capacity (ROS-induced QTLs) and the risk of developing IPA among HSCT recipients.

## 1 Introduction

The production of reactive-oxygen species (ROS) against microbial invaders is one of the early components of innate host defense [1]. The importance of this defense mechanism can be explained by the immediate and high production of ROS upon microbial invasion, and its small molecular size that allows transmembrane diffusion. Phagocytes produce ROS at the site of infection *via* the activation of nicotinamide adenine dinucleotide phosphate (NADPH) oxidases (NOXs), to kill pathogens, including fungi, that cannot be phagocytosed. NADPH oxidase and ROS production are also critical for the intracellular killing of fungi, either directly or indirectly [2]. In particular, neutrophils produce the majority of extracellular ROS, which also produce an enzyme, myeloperoxidase (MPO), that converts H_2_O_2_ to HOCl, which in turn contributes to the host defense against fungi. Upon oxidative stress, pathogens suffer cell injury, leading to DNA mutations [3], that result in gene expression and histone post-translational modifications [4] as well as lipid peroxidation [5].

The significance of the host oxidative immune response against fungal pathogens is highlighted by the susceptibility of patients deficient in this response [6]. Interestingly, early studies have reported that neutrophil microbicidal defect in chronic granulomatous disease (CGD) patients has been associated with the absence of respiratory burst activity, indicating its essential role in killing of the pathogens. CGD is a rare genetic disease, predisposing people to a life-long susceptibility to severe fungal infections [6] (especially *Aspergillus* spp.) and bacterial (*Staphylococcus aureus*) infections. Invasive fungal infections are a major cause of morbidity and mortality, especially among transplant recipients, with invasive candidiasis being the most common among liver-transplant recipients [7], and aspergillosis among stem-cell transplant recipients [8]. This increased susceptibility to invasive fungal infections point to the significance of ROS for fungal killing. Therefore, a better understanding of the factors that regulate ROS response, including genetic variants, could have an impact on the treatment of patients with invasive fungal infections.

Given that candidiasis and aspergillosis are infectious diseases with high mortality rates, especially among transplant recipients, the investigation of risk loci that determine the immune defense mechanisms against these fungal infections, and particularly of ROS production, is of great importance. However, GWAS studies alone cannot reveal the complexity of this interaction, and alternative approaches are needed to explain how genetic variants affect ROS response in the context of fungal diseases. Quantitative trait locus (QTL) mapping is a tool that can offer insight into the complex interplay between genes and phenotype. It can help to prioritize genetic variants that regulate ROS production, and in turn potentially cause disease. Therefore, we assessed the impact of genome-wide genetic polymorphisms on ROS production in response to *Candida albicans* (*C. albicans*) and *Aspergillus fumigatus* (*A. fumigatus*) using a population-based cohort of Western European ancestry, the 200FG cohort [9]. To investigate how the identified ROS-QTLs influence the risk of fungal infections, we investigated their impact on the risk of invasive pulmonary aspergillosis (IPA) among a hematopoietic stem-cell transplant (HSCT) patient cohort. These findings can help uncover the genetic basis of ROS production and its influence on disease susceptibility, which ultimately can help design novel therapeutic strategies.

## 2 Materials and methods

### 2.1 Ethics statement

The Human Functional Genomics Project (HFGP) (http://www.humanfunctionalgenomics.org) was approved by the Ethical Committee of Radboud University Nijmegen, the Netherlands (nr. 42561.091.12). Experiments were conducted according to the principles expressed in the Declaration of Helsinki. Samples of venous blood were drawn after informed consent was obtained.

Approval for the study using data from the transplant patients was obtained from the Ethics Committee for Research in Life and Health Sciences (CEICVS) of the University of Minho (125/014), the Ethics Committee for Health of the Instituto Português de Oncologia – Porto (26/015), the Ethics Committee of the Lisbon Academic Medical Center (632/014), and the National Commission for the Protection of Data, Portugal (1950/015). Written informed consent was obtained from the patient or a legal representative prior to collection of samples.

### 2.2 Population cohort

We investigated the role of genetic variations in ROS production in response to *C. albicans* and *A. fumigatus* infection in a population-based cohort: the 200 Functional Genomics cohort (200FG). The cohort comprises 197 healthy adults working as foresters from the ‘Geldersch Landschap’, ‘Hoge Veluwe’, ‘Twickel’, and ‘Kroondomein het Loo’ in the Netherlands. These individuals were between 23 and 73 years old, and 77% were male, and 23% female.

For the genetic association study, a total of 386 hematological patients of European ancestry undergoing allogeneic HSCT at Instituto Português de Oncologia, Porto, and at Hospital de Santa Maria, Lisbon (Portugal), were enrolled between 2009 and 2016. The demographic and clinical characteristics of the patients are summarized in Supplementary Table 1. Ninety-nine cases of probable/proven IPA were identified according to the 2008 criteria from the European Organization for Research and Treatment of Cancer/Mycology Study Group (EORTC/MSG) [10]. Patients with a diagnosis of “possible” infection or pre-transplant fungal infection were excluded from the study. Seven patients with pre-transplant fungal infection were also removed from analyses.

### 2.3 DNA isolation and SNP genotyping

Genomic DNA was isolated from whole blood of patients using the QIAcube automated system (Qiagen). Genotyping was performed using KASPar assays (LGC Genomics) in an Applied Biosystems 7500 Fast Real-Time PCR system (Thermo Fisher Scientific), according to the manufacturer's instructions. Quality control for the genotyping results was achieved with negative controls and randomly selected samples with known genotypes.

### 2.4 PBMC collection & stimulation experiments

Peripheral blood mononuclear cell (PBMC) collection and stimulation experiments using fungal stimuli were previously described [9]. Briefly, after obtaining the volunteer's written informed consent, samples of venous blood were drawn into 10 mL EDTA Monoject tubes (Medtronic, Dublin). PBMCs were separated by density centrifugation of EDTA blood diluted 1:1 in pyrogen-free saline solution over Ficoll-Paque (Pharmacia Biotech, Uppsala). The separated cell fraction was washed twice in saline and suspended in RPMI 1640 medium supplemented with gentamicin (50 μg/mL), l-glutamine (2 mM) and pyruvate (1 mM). The cells were counted in a Coulter counter (Beckman Coulter, Pasadena), and the concentration was adjusted to 5 × 10^6^ cells/mL.

### 2.5 Fungal culture

*C. albicans* (ATCC MYA-3573/UC 820) was grown from glycerol stock on Sabouraud dex-trose (SD) plates. For all experiments, a single colony was grown in Sabouraud broth at 37 °C for 12 h to log phase while shaking at 120 rpm. The harvested *C. albicans* yeast were washed twice in sterile phosphate buffered saline (PBS). *A. fumigatus* (V05-27) was grown from glycerol stock on Sabouraud dextrose agar for 7 days at 37 °C. Conidia were harvested in sterile PBS-tween-20 (0.1%), subsequently washed twice in sterile PBS. Harvested *C. albicans* yeast and *A. fumigatus* conidia were counted using a Bürker hemocytometer and adjusted to the desired concentration in RPMI 1640 Dutch modification (Gibco).

### 2.6 ROS production

Reactive oxygen species were measured in PBMCs of healthy volunteers from the 200FG cohort as previously described [11]. Upon stimulation, the ROS production of cells was measured by oxidation of luminol (5-amino-2,3, dihydro-1,4-phtalazinedione). PBMCs (5 × 10^5^) were suspended in Hanks Balanced Salt Solution (HBSS) and put in dark 96-well plates. Cells were exposed to 1 × 10^6^/mL *C. albicans* yeast and 1 × 10^7^/mL *A. fumigatus* conidia in the presence of 10% autologous serum for opsonization, with 20 μL of 1 mM luminol (final concentration 50 μM). Chemiluminescence was measured in BioTek Synergy HTreader at 37 °C for 1 h with intervals of 2.23 min.

### 2.7 Genotyping, quality control, imputation

DNA samples of individuals from the 200FG cohort were genotyped using the commercially available SNP chip, Illumina HumanOmniExpressExome-8 v1.0. The genotype calling was performed using Optical 0.7.7 [12] using the default settings. Standard pre-imputation genotype data quality control was performed, during which we excluded individuals with misconcordant sex information, ±3 standard deviations from mean heterozygosity and related samples with a PIHAT >0.2. Furthermore, samples with a call rate ≤0.9 were excluded from the dataset, as were variants with call rate ≤0.9 and minor allele frequency (MAF) ≤ 0.05. This resulted in a dataset of 280 samples containing genotype information on 125,427 variants. Imputation was performed using the haplotype reference consortium (HRC r1.1 2016) as a reference panel [13] using the Michigan Imputation Server (https://imputationserver.sph.umich.edu/) [14]. Data were phased using Eagle version 2.4. Thereafter, single nucleotide polymorphisms (SNPs) were removed based on following criteria: imputation quality score R^2^ < 0.3, MAF <0.05, and HWE< 10^−6^. In total 4,303,872 SNPs with MAF 5% were retained for follow-up QTL mapping.

### 2.8 ROS quantitative trait loci (QTL) mapping

Both genotype and ROS production data was available for 99 individuals out of 197 individuals. To check for normality of the ROS levels, we followed a visual inspection of the data using both raw and rank-based inverse transformed data. ROS levels followed non-Gaussian and Gaussian distribution before and after data transformation respectively (Fig. S1). Following quality check for the distribution of ROS levels and after excluding genetic outliers, we mapped the rank-based inverse-transformed ROS levels to genotype data using a linear regression model with age and sex as covariates to correct the distributions of ROS levels. Sex information was coded with 0 for females and 1 for males. QTL mapping was performed using the ROS levels in response to *C. albicans* and *A. fumigatus* independently. We used a cutoff of 1 × 10^−5^ to identify suggestive QTL associations affecting ROS levels in response to *C. albicans* and *A. fumigatus* (ROS-QTLs).

### 2.9 Statistical analysis

Statistical analysis and visualization using the genetic data were performed in R (version 4.1.2): a free software environment for statistical computing and graphics [15]. R-package Matrix-eQTL was used for QTL mapping [16], in which linear model was applied with age and sex as covariates. Quality control of the pre- and post-imputed genetic data was performed using PLINK (v1.90b6.18 64-bit) [17].

The probability of IPA according to donor ROS-QTL genotypes was determined using the cumulative incidence method and compared using Gray's test [18]. Cumulative incidences of infection at 24 months were computed with the cmprsk package for R version 2.10.1, with censoring of data at the date of last follow-up visit and relapse and death as competing events [19]. All clinical and genetic variables achieving a P value ≤ 0.15 in the univariate analysis were entered one by one in a pairwise model together and kept in the final model if they remained significant (*P* < 0.05). Multivariate analysis was performed using the subdistribution regression model of Fine and Gray with the cmprsk package for R version 2.10.1 [20].

## 3 Results

### 3.1 Identification of genetic variants that influence ROS production in response to fungal pathogens

**Fig. 1:**
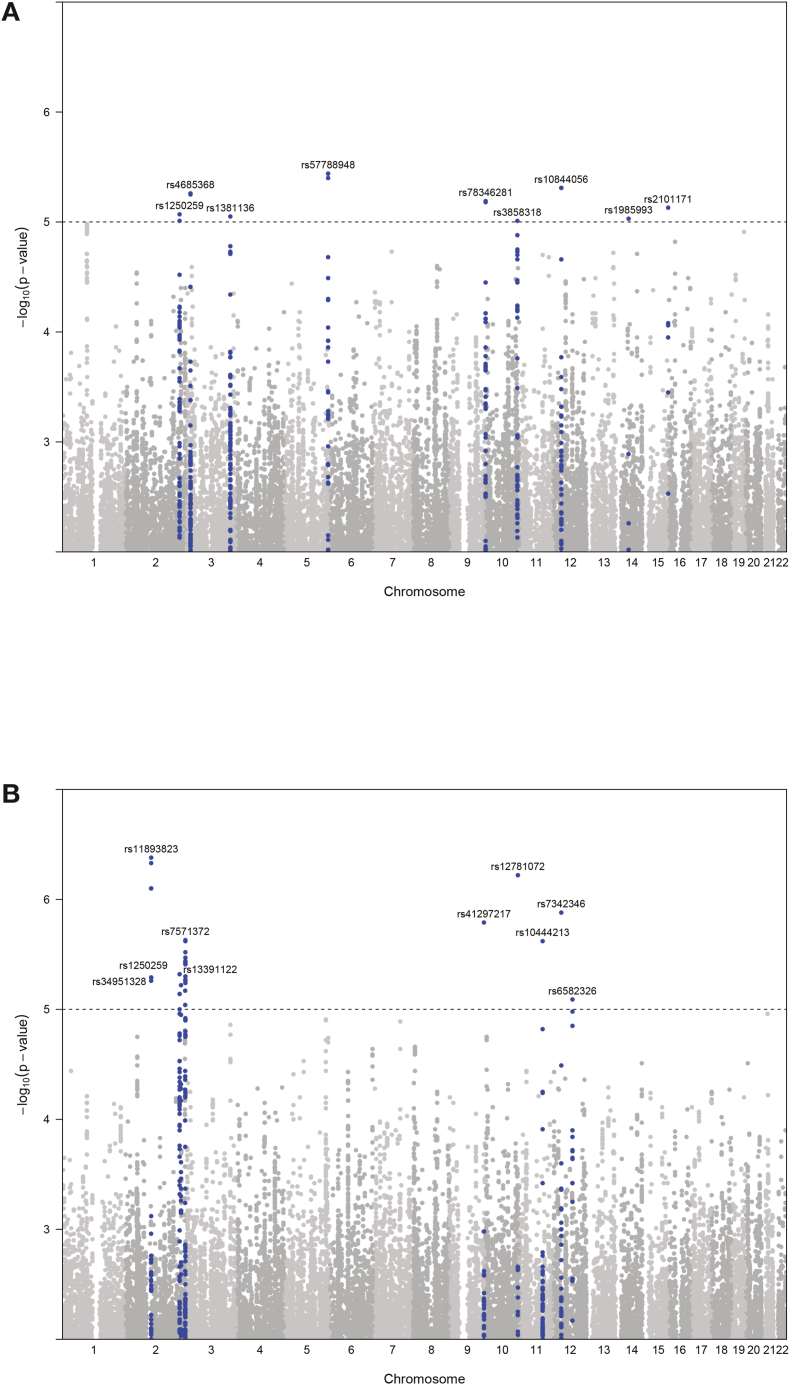
Manhattan plots showing the QTLs influencing ROS production upon (A) *C. albicans* and (B) *A. fumigatus* stimulation. Y-axis represents -log 10 *P*-values of SNPs. X-axis shows chromosomal positions. The dashed line indicates the suggestive *P* threshold for association (<9.99 × 10^−6^).

We first investigated whether host genetic variation affects the inter-individual differences in ROS production upon stimulation with the fungal pathogens *C. albicans* and *A. fumigatus*. Upon quality control of genetic and functional data, we obtained 99 samples with both genetic and ROS measurements. For QTL mapping, we selected SNPs that showed a minor allele frequency (MAF) ≥ 5% and passed other quality filters (see Materials and Methods). Using the ROS and genotype data, we mapped ROS-QTLs using a linear model with age and sex as covariates. QTL mapping revealed ten and nine suggestive, independent QTLs (*P* < 9.99 × 10^−6^) upon stimulation with *C. albicans* and *A. fumigatus*, respectively (Table 1 and Fig. 1). Among these 19 QTLs, two QTLs (rs1250259 on chromosome 2; and rs10844056 on chromosome 12, which is in strong LD [r^2^ = 1 and D' = 1] with rs7342346) were found to be associated with ROS production in response to both fungal pathogens. While most of these SNPs fall within intronic/intergenic regions, we found rs1250259 to be a missense variant within the fibronectin 1 (*FN1*) gene. *C. albicans* and *A. fumigatus* hyphae and conidia are known to adhere to fibronectin [21,22]. We also found another SNP, rs4685368 (chr3: 16681999), that showed QTL effect in response to both fungal pathogens, with a *P* value of 7.69 × 10^−5^ in response to *C. albicans* (not shown on Table 1), indicating the role of common genetic loci in regulating ROS production in response to fungal pathogens. These shared genetic loci suggest that the mechanisms regulating ROS production are shared between the two fungal pathogens. In line to this, we observed a strong correlation between ROS production in response to *C. albicans* and *A. fumigatus* (Fig. S2).

**Table 1:** ROS-QTL loci in response to *C. albicans* and *A. fumigatus* at *P* < 9.99 × 10^−6^.

Chr	Pos	SNP	Minor allele	Function	Fungus	P value	beta	Gene(s)
2	216300482	rs1250259	T	missense variant	*A. fumigatus*	8.43 x10^-6^	0.70	*ATIC*^*a*^*, FN1*^*b,c,d,e*^*, LINC00607*^*c*^*, ATIC*^*d*^
2	103581470	rs11893823	T	downstream gene variant	*C. albicans*	4.13 x10^-7^	−0.84	*MFSD9*^*a*^*, SLC9A4*^*b,c*^
2	239228138	rs7571372	G	upstream gene variant	*C. albicans*	2.32 x10^-6^	0.73	*AC016757.3*^*a*^*,TRAF3IP1*^*a*^*,HES6a,RAMP1*^*e*^*, PER2*^*d*^*, ASB1*^*c*^*, HES6*^*d*^*, ILKAP*^*d*^*, TRAF3IP1*^*d*^*, UBE2F*^*c*^
2	216300482	rs1250259	T	missense variant	*C. albicans*	4.83 x10^-6^	0.72	*ATIC*^*a*^*, ATIC*^*d*^*, FIN*^*b,c,d,e*^*, LINC00607*^*c*^
2	221804970	rs34951328	G	intergenic variant	*C. albicans*	6.06 x10^-6^	−0.87	*EPHA4*^*b,c*^
2	238506581	rs13391122	T	downstream gene variant	*C. albicans*	6.77 x10^-6^	−0.81	*LRRFIP1*^*a*^*, LRRFIP1*^*c,d,e*^*, COL6A3*^*c,d,e*^
3	16681999	rs4685368	A	intron variant	*A. fumigatus*	5.56 x10^-^6^^	0.85	*RFTN1*^*c*^*, DPH3*^*c*^*, OXNAD1*^*c*^*, PLCL2*^*c,d,e*^
3	174864481	rs1381136	A	intron variant	*A. fumigatus*	8.88 x10^-6^	−0.61	
5	174600507	rs57788948	C	intergenic variant	*A. fumigatus*	3.63 x10^-6^	0.80	*HRH2*^*c*^
9	136338580	rs41297217	A	downstream gene variant	*C. albicans*	1.61 x10^-6^	1.33	*CACFD1*^*a*^*,MED22*^*a*^*,ADAMTSL2*^*a*^*,SURF6*^*a*^*,RALGDS*^*a*^*, ADAMTS13*^*a*^*,SLC2A6*^*b,c,d*^*, SURF4*^*b,c,d,e*^*, RALGDS*^*c,d*^*, RPL7A*^*c,d,e*^*, GBGT1*^*c*^*, DBH-AS1*^*d*^*, SURF2*^*d*^*, SURF6*^*c,d*^*, SARDH*^*d*^*, ADAMTS13*^*e*^*, SURF1*^*c*^*, REXO4*^*d*^*, C9orf96*^*c*^
10	1988696	rs78346281	T	intergenic variant	*A. fumigatus*	6.46 x10^-6^	0.72	
10	127969267	rs3858318	G	intron variant	*A. fumigatus*	9.86 x10^-6^	−0.96	*ADAM12*^*d*^*, UROS*^*c*^*, BCCIP*^*d*^
10	130403853	rs12781072	A	intergenic variant	*C. albicans*	6.00 x10^-7^	0.83	*MKI67*^*d*^
11	93012957	rs10444213	T	intergenic variant	*C. albicans*	2.40 x10^-6^	−0.73	*SMCO4*^*c,d*^*, SLC36A4*^*c*^*, SLC36A4*^*e*^*, KIAA1731*^*d*^*, TAF1D*^*d*^*, C11orf54*^*d*^*, SMCO4*^*e*^*, C11orf54*^*b*^
12	32074873	rs10844056	G	intergenic variant	*A. fumigatus*	4.90 x10^-6^	−0.61	*DDX11*^*a*^*, RP11-428G5.5*^*a*^*, RP11-467L13.5*^*a*^*, KIAA1551*^*a*^*, DENND5B*^*d*^*, BICD1*^*d*^*, DENND5B*^*c*^*, DENND5B*^*e*^*, METTL20*^*c*^*, KIAA1551*^*b*^
12	32074398	rs7342346	G	intergenic variant	*C. albicans*	1.31 x10^-6^	−0.65	*DDX11*^*a*^*, RP11-428G5.5*^*a*^*, RP11-467L13.5*^*a*^*, KIAA1551*^*a*^*, DENND5B*^*c,d,e*^*, BICD1*^*d*^*, METTL20*^*c*^*, KIAA1551*^*b*^
12	76417069	rs6582326	A	downstream gene variant	*C. albicans*	8.21 x10^-6^	−1.26	*PHLDA1*^*c,d,e*^*, BBS10*^*c*^*, NAP1L1*^*c,d*^*, OSBPL8*^*c,e*^
14	50510387	rs1985993	A	upstream gene variant	*A. fumigatus*	9.40 x10^-6^	−1.18	*METTL21D*^*a*^*, LINC01588/C14ORF182*^*b*^*, KLHDC2*^*c*^*, ARF6*^*c,d*^*, KLHDC1*^*d*^*, RPS29*^*c*^*, RPL3*6AL^b*,d*^*, KLHDC1*^*c*^*, C14orf182*^*c*^
15	101807915	rs2101171	C	downstream gene variant	*A. fumigatus*	7.39 x10^-6^	0.83	*VIMP*^*a*^*, CHSY1*^*a*^*, CHSY1*^*b,c*^*, VIMP*^*b,c,d,e*^*, LRRK1*^*d,e*^*,TARSL2*^*c*^*, PCSK6*^*c,e*^*, SNRPA1*^*c*^

Abbreviations: Chr, chromosome; Pos, chromosomal position in base-pairs.

^a^ Expression QTL effects in whole blood show a correlation between the ROS QTL SNP and the expression of that gene.

^b^ Gene is differentially expressed in response to 4-h stimulation with *C. albicans* (adjusted *P* value < 0.05).

^c^ Gene is differentially expressed in response to 24-h stimulation with *C. albicans* (adjusted *P* value < 0.05).

^d^ Gene is differentially expressed in response to 4-h stimulation with *A. fumigatus* (adjusted *P* value < 0.05).

^e^ Gene is differentially expressed in response to 24-h stimulation with *A. fumigatus* (adjusted *P* value < 0.05).

### 3.2 Prioritization of genes at QTL loci that affect ROS abundance in response to fungal pathogens

To prioritize potential causal genes at the strongly associated independent QTL loci (*P* < 9.99 × 10^−6^), two approaches were followed (Table 1). In the first approach, given that most of the SNPs were intronic and intergenic, which could have an effect on gene expression, we tested whether the ROS-QTLs affect the expression of genes in whole blood by using the largest, publicly available *cis*- and *trans*-expression QTL study in blood from a total of 31,684 individuals through the eQTLGen consortium (http://www.eqtlgen.org/index.html) [23]. Our strategy prioritized several interesting genes, including TRAF3 interacting protein 3 (*TRAF3IP1*) on chromosome 2, and two members of the ADAMTS (A Disintegrin and Metalloproteinase with Thrombospondin motifs) family on chromosome 9, *ADAMTS13* and *ADAMTSL2*. In addition to the intronic and intergenic variants, SNP rs1250259 on chromosome 2 is a missense variant mapped to the *FN1* gene which encodes fibronectin, a glycoprotein that is involved in cell adhesion and migration processes as well as blood coagulation and host defense [24]. Of note, this SNP was found to influence the expression levels of *ATIC* gene, which encodes a bifunctional enzyme that catalyzes the last two steps of purine biosynthesis [25,26], which is dependent of folic acid derivatives [27]. Second, we tested whether genes that are located within 1 MB window around the top ROS-QTLs were differentially expressed in response to *C. albicans* yeast or *A. fumigatus* conidia stimulation of PBMCs for 24 h, using our previously published transcriptomic dataset in response to *C. albicans* and *A. fumigatus* generated from eight healthy donors (Table 1 and Supplementary Table 2) [28]. Several of these genes are known to be involved in various processes, such as metabolic processes and cell communication. For example, *ARF6* and *RALGDS* are involved in phospholipase D signalling pathway, *GBGT1* in glycosphingolipid biosynthesis, and *CHSY1* in glycosaminoglycan biosynthesis. Two additional differentially expressed genes are involved in extracellular matrix (ECM)-receptor interaction, namely, *COL6A3* and *FN1*. Interestingly, *ATIC* gene is involved in antifolate resistance.

### 3.3 Impact of ROS QTLs on cytokine production in response to fungal pathogens

In addition, given the role of ROS in inflammatory regulation [29], we tested whether ROS-QTLs can act as cytokine-QTLs (cQTLs) in the context of *C. albicans* or *A. fumigatus* infection (Supplementary Table 3). For this, we used our previously published cQTL dataset in which genetic and *C. albicans* or *A. fumigatus* induced cytokine data generated from PBMCs isolated from healthy volunteers were used to map cQTLs in the context of fungal infection [9]. We found that five ROS-QTL loci influence one or two of the monocyte-derived (IL-6 and TNFα) or T-cell derived cytokines (IL-17 and IL-22) in response to *C. albicans* yeast or hyphae at a *P* value nominal significance <0.05. One locus at chromosome 5 (rs78346281) found to influence TNFα levels in response to *A. fumigatus*.

### 3.4 ROS-QTLs influence the risk of developing IPA among stem-cell transplant recipients

**Fig. 2:**
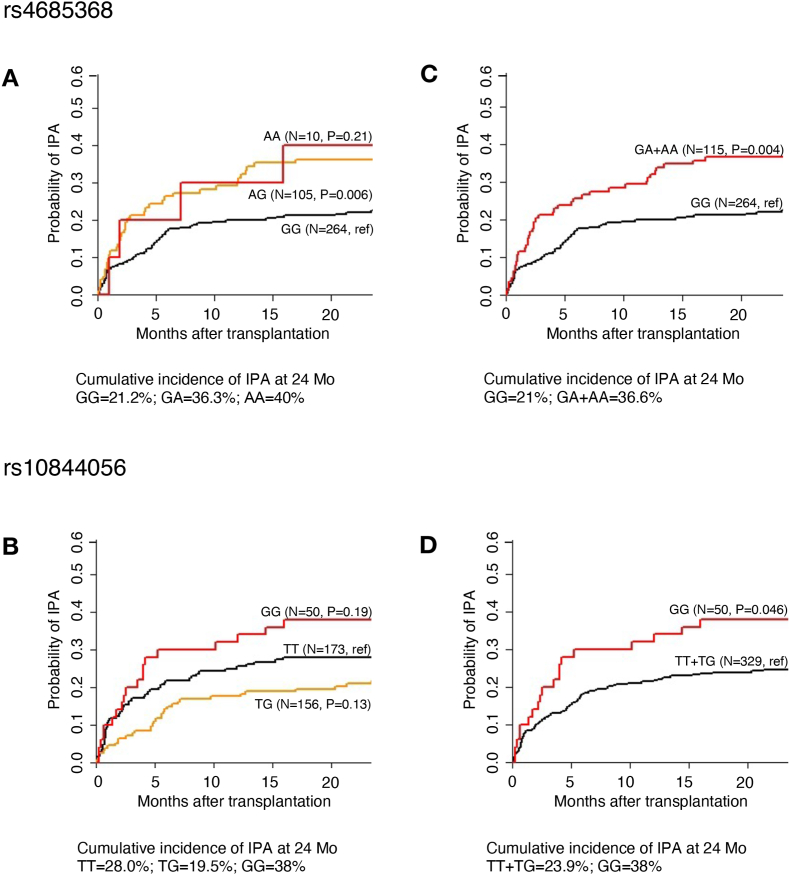
Cumulative incidence of IPA in recipients of allogeneic HSCT (n = 379) according to donor genotypes at (A) rs4685368 and (B) rs10844056. Results for rs4685368 and rs10844056 were also modeled using (C) dominant and (D) recessive modes of inheritance, respectively. Data were censored at 24 months, and relapse and death were considered competing events.

We next investigated the relationship between ROS-QTLs and susceptibility to IPA in patients at-risk of severe fungal infections. For this, we assessed the cumulative incidence of IPA in patients undergoing allogeneic HSCT according to the donor-genotypes (representing the cells of the innate immune system post transplantation) of the selected SNPs. Out of the top five ROS-QTLs tested, rs4685368 and rs10844056 were associated with an increased risk of IPA after transplantation in univariate analyses (Fig. 2). The cumulative incidence of IPA for donor rs4685368 was 21% for GG, 36% for GA (*P* value = 0.006), and 40% for AA genotypes (*P* value = 0.21). These differences were maintained after modelling a dominant mode of inheritance (cumulative incidence for GG was 21% and for the GA + AA genotype combination was 36% (*P* value = 0.004). As for the rs10844056 SNP, association with risk was only detected after recessive modelling. In these conditions, the cumulative incidence of IPA was 24% for the TT + TG genotype combination and 38% for the GG genotype (*P* value = 0.046).

**Fig. 3:**
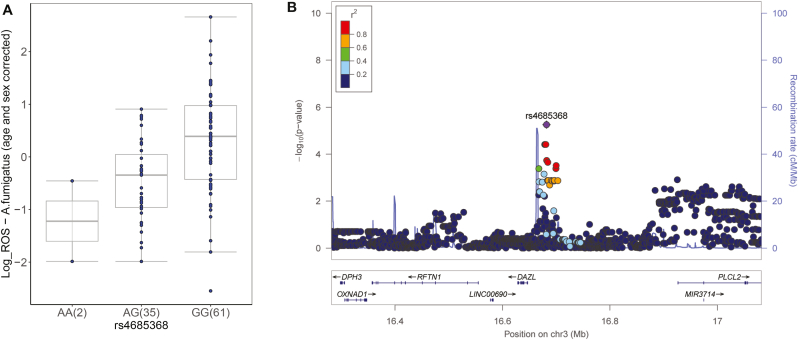
(A) Genotype-stratified ROS levels upon *A. fumigatus* stimulation at rs4685368 which was associated with an increased risk of IPA after transplantation. ROS levels were corrected for age and sex effects using a linear model. (B) Regional association plots of ROS QTLs in a window of 1 MB around rs4685368 (purple diamond). Each dot represents a SNP, the y-axis represents the negative logarithm of P values of SNP associations, and the x-axis shows chromosomal positions on Genome build GRCh37. The linkage disequilibrium (LD) of neighboring SNPs with the top SNP is color-coded. (For interpretation of the references to color in this figure legend, the reader is referred to the Web version of this article.)

In a multivariate model accounting for patient age and sex, hematological malignancy, type of transplantation, conditioning regimen, acute graft-versus-host-disease grades III-IV, and antifungal prophylaxis, the donor GA + AA genotype combination at rs4685368 conferred a 1.8-fold (95% confidence interval, 1.22–2.71) increased risk of developing IPA (*P* value = 0.0036) (Table 2). The rs10844056 SNP failed to reach a statistically significant level when performing multivariate testing (*P* value = 0.11). Of note, rs4685368-G allele was associated with increased production of ROS upon *A. fumigatus* stimulation, suggesting that the presence of allele G can be protective against the development of IPA (Fig. 3A). This is in agreement with our observation that transplant recipients that were homozygotes for allele G showed the least cumulative incidence of IPA compared to homozygotes or heterozygotes for the alternative allele A, even after using a multivariate model. In addition, rs4685368 is an intronic variant located in a locus in which *PLCL2* (inactive phospholipase C-like protein 2) gene is significantly differentially expressed upon *A. fumigatus* stimulation in PBMCs (adjusted *P* value < 0.05) (Fig. 3B). Collectively, these results highlight ROS-QTLs, and particularly rs4685368, as novel risk factors regulating susceptibility to IPA in HSCT recipients.

**Table 2:** Multivariate analysis of the association of ROS QTLs with the risk of invasive pulmonary aspergillosis among transplant recipients.

Genetic/clinical variables	Adjusted HR† (95% CI)	*P* value
Donor GA + AA genotype at rs4685368	1.81 (1.22–2.71)	0.0036
Donor GG genotype at rs10844056	1.50 (0.91–2.46)	0.110
Matched unrelated donor	1.66 (1.04–2.64)	0.033

HR, hazard ratio; CI, confidence interval. Multivariate analyses were based on the sub-distribution regression model of Fine and Gray. †Hazard ratios were adjusted for patient age and sex, hematological malignancy, type of transplantation, conditioning regimen, acute GVHD III-IV and antifungal prophylaxis. Only the clinical variables remaining significant after adjustment are shown.

## 4 Discussion

Given that oxidative burst is a hallmark of innate immunity, understanding how it is regulated during microbial invasion, including by fungal pathogens, is of great importance. In a given at-risk population, not all patients will develop infection, indicating that there is a strong genetic influence on susceptibility to infection. In line with this, we assessed the impact of genetic factors on ROS production capacity in a mixture of immune cells upon infection with the two most commonly encountered human fungal pathogens, *C. albicans* and *A. fumigatus*.

We identified several genetic loci that influence ROS production in response to *A. fumigatus* or *C. albicans*. These loci harbor genes that not only indicate an important role of ROS in immune responses, but also showing the potential of identifying novel targets to tailor immune responses in humans. For example, *TRAF3IP1* (TRAF3 interacting protein; also known as MIP-T3) gene from rs7571372 locus on chromosome 2 encodes a protein that is an inhibitor of the type I IFN response [30], which has been previously shown as a central host defence mechanism against *C. albicans* [31,32] and *A. fumigatus* [33]. Of note, a previous study demonstrated the potential clinical use of IFN-γ (type II IFN) as adjunctive immunotherapy to partially restore immune function in fungal sepsis patients [34]. It has been previously shown that drug mechanisms supported by genetic evidence would succeed twice as often as those without (from phase I to approval) [35]. Thus, administration of IFN-γ combined with patient stratification based on SNP genotype warrants future clinical studies to fully assess its potential clinical benefit in fungal sepsis patients.

Another interesting locus on chromosome 2 harbours rs1250259, which is a missense variant mapped to the *FN1* gene that was found to influence ROS levels upon stimulation with both fungal pathogens. This gene encodes fibronectin, an important glycoprotein involved in various processes, such as cell adhesion, migration, blood coagulation and host defense [24]. ROS have been described as essential mediator of cell adhesion [36], which is mediated by fibronectin, among other extracelular cell matrix (ECM) components. The role of fibronectin is to anchor cells to ECM *via* integrin binding [24]. *C. albicans* and *A. fumigatus* hyphae and conidia are known to adhere to fibronectin [[21], [22]]. *C. albicans* expresses adhesive proteins at the cell surface that interact with major ECM proteins, such as fibronectin [22], among other ECM components (laminin and vitronectin, collagen type IV) [[37], [38], [39]]. Inhaled *A. fumigatus* conidia have higher chances of attachement and invasion since diseased lungs have increased amounts of fibronectin and other ECM components [21]. In addition to fibronectin, two additional genes, *ADAM12* related to rs3858318 locus at chromosome 10 and *ADAMTSL2* related to rs41297217 locus at chromosome 9 seem to be implicated in ECM interactions [40]*. ADAM12* encodes a metalloprotease, a member of the ADAM (a disintegrin and metalloproteinasese) protein family, and it has been implicated in a variety of biological processes, involving cell-cell and cell-matrix interactions [41]. Also, *ADAM12* is upregulated in endothelial or epithelial cells in response to TNFα [42,43]. *ADAMTSL2* encodes another member of ADAMTS-like protein family (a disintegrin and metalloproteinase with thrombospondin motifs), which is a secreted glycoprotein that binds the cell surface and extracellular matrix, and it also interacts with latent transforming growth factor beta binding protein 1 [44]. All this evidence suggests a critical role of ECM components in fungal infections and their potential to serve as targets for the prevention and treatment of such infections. Targeting these molecules in the host or fungus could block the adhesion of fungi. However, further studies are needed to shed light on the role of ROS production in the adhesion of the fungal pathogens with fibronectin and other ECM components during infection.

In addition to the missense variant (rs1250259) described above, two more loci at chromosome 12 and 3 marked by the rs10844056 (chr12: 32074873), and rs4685368 (chr3: 16681999), respectively, showed QTL effect in response to both fungal pathogens. This finding suggests the critical role of ROS production against both pathogens despite their differences, with *Candida* being a commensal in the human gastrointestinal tract and *Aspergillus* being an airborne abundant fungus in the normal environment. Interestingly, none of the ROS QTLs were associated with candidaemia (Supplementary Table 4). The absence of major *Candida* infections in patients with chronic granulomatous disease due to ROS deficiency is a strong argument that in absence of ROS other immune mechanisms provide sufficient redundancy to mediate resistance to candidiasis. However, both SNPs found to significantly influence the risk of invasive pulmonary aspergillosis (IPA) among HSCT recipients. Given that invasive fungal infections (IFIs) cause significant morbidity and mortality in HSCT [45], this finding highlights not only the importance of patient stratification to identify those at high-risk of developing aspergillosis, but also indicates an important role of ROS in regulating immune response in these patients. Therefore, novel pharmacological options may help tailor the immune responses in human *via* ROS modulation [46].

There are also limitations to this study. First, the experimental set-up of *ex vivo* PBMC stimulation with the fungal pathogen did not fully mirror the physiological responses upon infection. However, this model provided the opportunity to study the interactions between immune cells, such as monocytes, T and B cells. Second, it would be interesting to investigate the effect of variants on the gene expression in the context of *C. albicans* or *A. fumigatus* stimulation in order to capture context-specific QTL effects that are not present in the publicly available eQTL datasets. Because of the relatively small sample size and the lack of a validation using an independent cohort, we cannot exclude the possibility that some of the QTLs were false positives. Nevertheless, we observed several shared loci that affect ROS production consistently in response to two different fungal pathogens, indicating potential genetic regulators of ROS production. In addition, we also provided evidence of the role of two SNPs (rs10844056 and rs4685368) in the increasing risk of IPA among HSCT recipients. In particular, there are evidence that *PLCL2* gene from rs4685368 locus has been associated with combined oxidative phosphorylation deficiency 9 [47] (OMIM: 614582). Combined oxidative phosphorylation deficiency is a rare autosomal recessive disorder with variable manifestations resulting from a defect in the mitochondrial oxidative phosphorylation. A defective mitochondrial oxidative phosphorylation may have detrimental impact on ROS production, regulation and response, which, in turn, will lead to a defective phagocytosis and clearance of the fungus. However, the role of *PLCL2* gene in mitochondrial dysfunction and the consequences on the ROS production capacity, and, by extend, to the increasing risk to a fungal infection, warrants further investigation.

Overall, the significance of ROS for fungal killing is clear, however, the factors that regulate ROS production and response, are of critical importance to better understand the host-pathogen interactions. Deciphering these factors could have an impact on the treatment of patients with invasive fungal infections. To this end, by applying a functional genomics approach in a population-based cohort, we provided evidence that genetic variation can influence ROS production capacity, and, importantly, the risk of developing IPA in stem-cell transplant recipients. In the future, stratifying patients based on the genetic profiling would allow us to identify those at high-risk, who will benefit most from a preventive or treatment strategy.

## Funding

This study was supported by the European Union's Horizon 2020 research and innovation programme under grant agreement no. 847507 (HDM-FUN). MGN was supported by an ERC Advanced grant (833247) and a Spinoza grant of the Netherlands Association for Scientific Research. VK was supported by a Research Grant [2017] of the European Society of Clinical Microbiology and Infectious Diseases (ESCMID) and Hypatia tenure track grant. AC was supported by the Fundação para a Ciência e a Tecnologia (FCT) (UIDB/50026/2020 and UIDP/50026/2020), the Northern Portugal Regional Operational Programme (NORTE 2020), under the Portugal 2020 Partnership Agreement, through the European Regional Development Fund (ERDF) (NORTE-01-0145-FEDER-000039), and the “la Caixa” Foundation (ID 100010434) and FCT under the agreement LCF/PR/HR17/52190003. CC was supported by FCT (CEECIND/04058/2018 and PTDC/SAU-SER/29,635/2017) and the Gilead Research Scholars Program – Antifungals. SMG was the recipient of a PhD fellowship funded by FCT (SFRH/BD/136,814/2018). MSG was supported by the German Research Foundation (Deutsche Forschungsgemeinschaft - DFG) Emmy Noether Program (project no. 434385622/GR 5617/1-1).

## Appendix A Supplementary data

Multimedia component 1 Multimedia component 1

Supplementary Fig. 1 Distribution plots of (A and B) raw and (C and D) rank-based inverse transformed ROS levels in response to (A and C) *C. albicans* and (B and D) *A. fumigatus*. Supplementary Fig. 1

Supplementary Fig. 2 Correlation between ROS levels in response to *C. albicans* and *A. fumigatus*. Data were rank-based inverse transformed and correlation was calculated using Spearman's rank correlation. Supplementary Fig. 2
