# Supplementary material for: Genetic determinants of fungi-induced ROS production are associated with the risk of invasive pulmonary aspergillosis
Source: Redox Biol. 2022 Jul 4;55:102391. doi: 10.1016/j.redox.2022.102391 (PMC9283926; doi:10.1016/j.redox.2022.102391)
Supplement: Multimedia component 1 [file mmc1.docx]

Supplementary Table 1. Baseline characteristics of transplant recipients enrolled in the study.

| **Variables** | **IPA**  **(n=99)** | **No IPA (n=287)** | **P value** |
| --- | --- | --- | --- |
| **Age at transplantation, no (%)** |  |  |  |
| ≤20 years | 8 (8.1) | 46 (16.0) | 0.143 |
| 21 – 40 years | 29 (29.3) | 79 (27.5) |  |
| >40 years | 62 (62.6) | 162 (56.5) |  |
| **Gender, no (%)** |  |  |  |
| Female | 43 (43.4) | 118 (41.1) | 0.680 |
| Male | 56 (56.6) | 169 (58.9) |  |
| **Underlying disease, no. (%)** |  |  |  |
| Acute leukemia | 54 (54.5) | 150 (52.3) | 0.062 |
| Chronic lymphoproliferative diseases | 13 (13.1) | 64 (22.3) |  |
| Myelodysplastic/myeloproliferative diseases | 15 (15.2) | 21 (7.3) |  |
| Chronic myeloproliferative diseases | 8 (8.1) | 19 (6.6) |  |
| Aplastic anemia | 6 (6.1) | 13 (4.5) |  |
| Other | 3 (3.0) | 20 (7.0) |  |
| **Transplantation type, no. (%)** |  |  |  |
| Matched, related | 30 (30.3) | 141 (49.1) | <0.001 |
| Matched, unrelated | 44 (44.4) | 72 (25.1) |  |
| Mismatched, related | 0 (0.0) | 5 (1.7) |  |
| Mismatched, unrelated | 25 (25.3) | 69 (24.0) |  |
| **Graft source, no. (%)** |  |  |  |
| Peripheral blood | 81 (81.8) | 237 (82.6) | 0.249 |
| Bone-marrow | 18 (18.2) | 43 (15.0) |  |
| Cord blood | 0 (0.0) | 7 (2.4) |  |
| **Disease stage, no. (%)** |  |  |  |
| First complete remission | 50 (50.5) | 156 (54.4) | 0.787 |
| Second or subsequent remission, or relapse | 19 (19.2) | 51 (17.8) |  |
| Active disease | 30 (30.3) | 80 (27.9) |  |
| **Conditioning regimen, no (%)** |  |  |  |
| RIC | 70 (70.7) | 176 (59.9) | 0.103 |
| Myeloablative | 29 (29.3) | 111 (38.7) |  |
| **CMV serostatus of donor and recipient, no. (%)** |  |  |  |
| D-/R+ or D+/R+ | 86 (86.9) | 260 (90.6) | 0.297 |
| D-/R- or D+/R- | 13 (13.1) | 27 (9.4) |  |
| **Duration of neutropenia, mean days (range)†** | 12.8 (8 – 39) | 13.4 (2 – 35) | 0.301 |
| **Acute GVHD, no. (%)** |  |  |  |
| No GVHD or grades I – II | 75 (69.4) | 275 (87.4) | 0.016 |
| Grades III – IV | 24 (30.6) | 44 (12.6) |  |
| **Antifungal prophylaxis, no. (%)‡** |  |  |  |
| Fluconazole | 55 (55.6) | 115 (40.1) | 0.011 |
| Posaconazole | 22 (22.2) | 79 (27.5) |  |
| Other | 7 (7.1) | 12 (4.2) |  |
| None or unknown | 15 (15.1) | 81 (28.2) |  |

Chronic lymphoproliferative diseases included cases of chronic lymphocytic leukemia, multiple myeloma, and B- and T-cell lymphomas. Chronic myeloproliferative diseases included cases of chronic myelogenous leukemia and primary myelofibrosis. Other diseases included cases of idiopathic medullar aplasia, lymphohistiocytosis, hemoglobinopathies and paroxysmal nocturnal hemoglobinuria. RIC, reduced intensity conditioning; CMV, cytomegalovirus; D, donor; R, recipient; GVHD, graft-versus-host-disease. †Neutropenia was defined as ≤0.5×10^9^ cells/L. ‡Other antifungals used in prophylaxis included voriconazole, liposomal amphotericin B, itraconazole and caspofungin. P values were calculated by Fisher’s exact probability t-test or Student’s t-test for continuous variables.

Supplementary Table 2. Summary statistics of differentially expressed genes from the top QTL loci upon stimulation with *C. albicans* or *A. fumigatus* (adjusted *P* value < 0.05). Data were previously reported by *Bruno M, et al*^28^.

| **SNP** | **Gene** | **baseMean** | **Log2FoldChange** | **P adjusted** | **Stimulation** | **QTL** |
| --- | --- | --- | --- | --- | --- | --- |
| rs11893823 | *SLC9A4* | 3.48 | 2.83 | 2.86E-04 | *Candida_24h* | Candida_specific |
| rs11893823 | *SLC9A4* | 7.12 | 1.50 | 2.55E-02 | *Candida_4h* | Candida_specific |
| rs41297217 | *C9orf96* | 10.15 | -0.81 | 4.74E-02 | *Candida_24h* | Candida_specific |
| rs13391122 | *COL6A3* | 12.25 | -1.41 | 9.02E-04 | *Aspergillus_4h* | Candida_specific |
| rs41297217 | *ADAMTS13* | 21.11 | -0.78 | 2.04E-02 | *Aspergillus_24h* | Candida_specific |
| rs7342346 | *METTL20* | 34.36 | 0.49 | 4.10E-02 | *Candida_24h* | Candida_specific |
| rs13391122 | *COL6A3* | 41.74 | -1.01 | 4.58E-05 | *Candida_24h* | Candida_specific |
| rs13391122 | *COL6A3* | 46.31 | -0.52 | 2.50E-02 | *Aspergillus_24h* | Candida_specific |
| rs7571372 | *RAMP1* | 48.48 | -1.32 | 1.41E-07 | *Aspergillus_24h* | Candida_specific |
| rs7571372 | *HES6* | 48.66 | 0.73 | 4.75E-04 | *Aspergillus_4h* | Candida_specific |
| rs12781072 | *MKI67* | 68.08 | -0.65 | 1.31E-03 | *Aspergillus_4h* | Candida_specific |
| rs7342346 | *BICD1* | 86.04 | -0.69 | 1.95E-04 | *Aspergillus_4h* | Candida_specific |
| rs41297217 | *DBH-AS1* | 87.88 | -0.53 | 2.76E-03 | *Aspergillus_4h* | Candida_specific |
| rs10444213 | *SMCO4* | 93.66 | 0.68 | 2.85E-02 | *Aspergillus_24h* | Candida_specific |
| rs10444213 | *SMCO4* | 103.13 | -0.78 | 1.87E-06 | *Aspergillus_4h* | Candida_specific |
| rs7342346 | *DENND5B* | 111.59 | -0.81 | 1.01E-07 | *Aspergillus_4h* | Candida_specific |
| rs41297217 | *SARDH* | 133.08 | -0.42 | 4.61E-03 | *Aspergillus_4h* | Candida_specific |
| rs41297217 | *SURF2* | 137.55 | 0.40 | 3.07E-03 | *Aspergillus_4h* | Candida_specific |
| rs41297217 | *GBGT1* | 164.86 | 0.97 | 2.52E-07 | *Candida_24h* | Candida_specific |
| rs7571372 | *TRAF3IP1* | 166.97 | -0.36 | 9.37E-03 | *Aspergillus_4h* | Candida_specific |
| rs7342346 | *DENND5B* | 171.37 | 0.37 | 1.68E-02 | *Aspergillus_24h* | Candida_specific |
| rs7342346 | *DENND5B* | 176.54 | 0.40 | 8.23E-03 | *Candida_24h* | Candida_specific |
| rs6582326 | *BBS10* | 179.38 | -0.41 | 2.24E-03 | *Candida_24h* | Candida_specific |
| rs10444213 | *SMCO4* | 192.98 | 2.07 | 2.71E-08 | *Candida_24h* | Candida_specific |
| rs7571372 | *UBE2F* | 217.84 | 0.27 | 4.55E-02 | *Candida_24h* | Candida_specific |
| rs41297217 | *SURF1* | 304.58 | -0.27 | 2.12E-02 | *Candida_24h* | Candida_specific |
| rs41297217 | *SURF6* | 321.06 | -0.36 | 3.10E-03 | *Candida_24h* | Candida_specific |
| rs6582326 | *PHLDA1* | 321.06 | 0.60 | 4.01E-04 | *Aspergillus_24h* | Candida_specific |
| rs10444213 | *C11orf54* | 322.11 | -0.26 | 3.09E-02 | *Candida_4h* | Candida_specific |
| rs10444213 | *C11orf54* | 347.13 | -0.23 | 2.37E-02 | *Aspergillus_4h* | Candida_specific |
| rs41297217 | *REXO4* | 363.04 | 0.22 | 3.31E-02 | *Aspergillus_4h* | Candida_specific |
| rs6582326 | *PHLDA1* | 378.69 | 0.80 | 7.65E-07 | *Candida_24h* | Candida_specific |
| rs34951328 | *EPHA4* | 413.11 | -0.64 | 4.62E-05 | *Candida_24h* | Candida_specific |
| rs41297217 | *SURF6* | 439.42 | 0.25 | 1.06E-02 | *Aspergillus_4h* | Candida_specific |
| rs10444213 | *SLC36A4* | 472.75 | 0.36 | 1.24E-03 | *Aspergillus_24h* | Candida_specific |
| rs7571372 | *PER2* | 511.50 | 0.45 | 2.17E-06 | *Aspergillus_4h* | Candida_specific |
| rs10444213 | *SLC36A4* | 539.53 | 0.56 | 4.14E-04 | *Candida_24h* | Candida_specific |
| rs7571372 | *ILKAP* | 585.15 | 0.23 | 6.75E-03 | *Aspergillus_4h* | Candida_specific |
| rs10444213 | *KIAA1731* | 626.30 | -0.26 | 6.01E-03 | *Aspergillus_4h* | Candida_specific |
| rs34951328 | *EPHA4* | 649.74 | -0.46 | 9.81E-03 | *Candida_4h* | Candida_specific |
| rs7571372 | *ASB1* | 745.64 | -0.37 | 2.15E-04 | *Candida_24h* | Candida_specific |
| rs41297217 | *SLC2A6* | 900.39 | 0.41 | 3.85E-03 | *Aspergillus_4h* | Candida_specific |
| rs41297217 | *SLC2A6* | 934.34 | 2.14 | 1.27E-24 | *Candida_24h* | Candida_specific |
| rs41297217 | *SLC2A6* | 1549.21 | 1.77 | 3.05E-06 | *Candida_4h* | Candida_specific |
| rs41297217 | *SURF4* | 1573.02 | 0.30 | 1.76E-02 | *Aspergillus_24h* | Candida_specific |
| rs10444213 | *TAF1D* | 1739.66 | 0.24 | 7.83E-03 | *Aspergillus_4h* | Candida_specific |
| rs41297217 | *SURF4* | 1809.55 | 0.62 | 3.08E-11 | *Candida_24h* | Candida_specific |
| rs6582326 | *PHLDA1* | 1889.18 | 0.92 | 4.29E-07 | *Aspergillus_4h* | Candida_specific |
| rs13391122 | *LRRFIP1* | 2034.25 | 0.33 | 1.68E-04 | *Aspergillus_24h* | Candida_specific |
| rs6582326 | *OSBPL8* | 2109.10 | 0.31 | 2.00E-02 | *Aspergillus_24h* | Candida_specific |
| rs13391122 | *LRRFIP1* | 2113.59 | 0.39 | 3.07E-08 | *Candida_24h* | Candida_specific |
| rs6582326 | *OSBPL8* | 2171.69 | 0.33 | 2.30E-02 | *Candida_24h* | Candida_specific |
| rs41297217 | *RPL7A* | 2255.25 | -0.50 | 8.33E-08 | *Candida_24h* | Candida_specific |
| rs41297217 | *RPL7A* | 2384.92 | -0.24 | 1.08E-02 | *Aspergillus_24h* | Candida_specific |
| rs41297217 | *RALGDS* | 2492.21 | 0.54 | 1.66E-04 | *Candida_24h* | Candida_specific |
| rs13391122 | *LRRFIP1* | 2564.35 | 0.18 | 1.80E-02 | *Aspergillus_4h* | Candida_specific |
| rs41297217 | *SURF4* | 3266.37 | 0.30 | 3.39E-04 | *Candida_4h* | Candida_specific |
| rs41297217 | *RALGDS* | 3329.00 | 0.62 | 6.54E-11 | *Aspergillus_4h* | Candida_specific |
| rs41297217 | *SURF4* | 3536.05 | 0.42 | 6.33E-08 | *Aspergillus_4h* | Candida_specific |
| rs41297217 | *RPL7A* | 3554.99 | 0.19 | 2.15E-02 | *Aspergillus_4h* | Candida_specific |
| rs6582326 | *NAP1L1* | 4928.61 | -0.32 | 4.62E-03 | *Candida_24h* | Candida_specific |
| rs6582326 | *NAP1L1* | 6526.88 | 0.21 | 3.66E-03 | *Aspergillus_4h* | Candida_specific |
| rs1250259 | *LINC00607* | 3.34 | -3.88 | 1.40E-05 | *Candida_24h* | Aspergillus_specific/Candida_specific |
| rs1250259 | *FN1* | 390.58 | -2.45 | 1.19E-18 | *Aspergillus_4h* | Aspergillus_specific/Candida_specific |
| rs1250259 | *ATIC* | 406.29 | -0.32 | 9.79E-04 | *Aspergillus_4h* | Aspergillus_specific/Candida_specific |
| rs1250259 | *FN1* | 448.73 | -1.12 | 2.07E-17 | *Candida_4h* | Aspergillus_specific/Candida_specific |
| rs1250259 | *FN1* | 1963.47 | -6.62 | 1.94E-76 | *Candida_24h* | Aspergillus_specific/Candida_specific |
| rs1250259 | *FN1* | 2272.29 | -2.85 | 5.80E-06 | *Aspergillus_24h* | Aspergillus_specific/Candida_specific |
| rs10844056 | *KIAA1551* | 7020.34 | -0.22 | 4.96E-02 | *Candida_4h* | Aspergillus_specific/Candida_specific |
| rs2101171 | *PCSK6* | 5.91 | 1.10 | 3.87E-02 | *Candida_24h* | Aspergillus_specific |
| rs2101171 | *PCSK6* | 9.26 | 1.93 | 1.20E-04 | *Aspergillus_24h* | Aspergillus_specific |
| rs3858318 | *ADAM12* | 27.35 | -1.37 | 2.03E-06 | *Aspergillus_4h* | Aspergillus_specific |
| rs10844056 | *METTL20* | 34.36 | 0.49 | 4.10E-02 | *Candida_24h* | Aspergillus_specific |
| rs1985993 | *KLHDC1* | 48.59 | -0.91 | 6.89E-05 | *Aspergillus_4h* | Aspergillus_specific |
| rs10844056 | *BICD1* | 86.04 | -0.69 | 1.95E-04 | *Aspergillus_4h* | Aspergillus_specific |
| rs1985993 | *KLHDC1* | 91.83 | -0.47 | 7.89E-03 | *Candida_24h* | Aspergillus_specific |
| rs57788948 | *HRH2* | 97.63 | 0.78 | 2.12E-03 | *Candida_24h* | Aspergillus_specific |
| rs1985993 | *LINC01588/C14ORF182* | 98.01 | 0.94 | 1.03E-12 | *Candida_4h* | Aspergillus_specific |
| rs10844056 | *DENND5B* | 111.59 | -0.81 | 1.01E-07 | *Aspergillus_4h* | Aspergillus_specific |
| rs1985993 | *C14orf182* | 111.90 | -0.33 | 3.47E-02 | *Candida_24h* | Aspergillus_specific |
| rs10844056 | *DENND5B* | 171.37 | 0.37 | 1.68E-02 | *Aspergillus_24h* | Aspergillus_specific |
| rs10844056 | *DENND5B* | 176.54 | 0.40 | 8.23E-03 | *Candida_24h* | Aspergillus_specific |
| rs3858318 | *UROS* | 182.08 | -0.34 | 6.58E-03 | *Candida_24h* | Aspergillus_specific |
| rs2101171 | *LRRK1* | 186.76 | -0.56 | 4.30E-02 | *Candida_4h* | Aspergillus_specific |
| rs2101171 | *LRRK1* | 191.66 | -0.80 | 5.41E-10 | *Aspergillus_4h* | Aspergillus_specific |
| rs2101171 | *TARSL2* | 224.04 | -0.64 | 7.01E-09 | *Candida_24h* | Aspergillus_specific |
| rs2101171 | *VIMP* | 269.65 | 0.29 | 3.05E-02 | *Aspergillus_24h* | Aspergillus_specific |
| rs3858318 | *BCCIP* | 333.19 | 0.29 | 8.61E-03 | *Aspergillus_4h* | Aspergillus_specific |
| rs2101171 | *VIMP* | 341.36 | 0.82 | 4.56E-12 | *Candida_24h* | Aspergillus_specific |
| rs2101171 | *SNRPA1* | 364.86 | -0.31 | 3.90E-03 | *Candida_24h* | Aspergillus_specific |
| rs4685368 | *DPH3* | 368.92 | 0.74 | 1.04E-11 | *Candida_24h* | Aspergillus_specific |
| rs4685368 | *OXNAD1* | 370.31 | -0.76 | 2.31E-08 | *Candida_24h* | Aspergillus_specific |
| rs2101171 | *VIMP* | 372.94 | 0.21 | 4.45E-02 | *Aspergillus_4h* | Aspergillus_specific |
| rs2101171 | *VIMP* | 386.84 | 0.43 | 2.06E-03 | *Candida_4h* | Aspergillus_specific |
| rs1985993 | *KLHDC2* | 456.33 | -0.54 | 6.67E-07 | *Candida_24h* | Aspergillus_specific |
| rs2101171 | *CHSY1* | 459.54 | 0.94 | 9.33E-16 | *Candida_24h* | Aspergillus_specific |
| rs4685368 | *PLCL2* | 671.59 | -0.62 | 1.86E-08 | *Aspergillus_4h* | Aspergillus_specific |
| rs2101171 | *CHSY1* | 767.62 | 0.52 | 3.76E-04 | *Candida_4h* | Aspergillus_specific |
| rs4685368 | *PLCL2* | 850.43 | -0.25 | 2.66E-02 | *Aspergillus_24h* | Aspergillus_specific |
| rs4685368 | *PLCL2* | 851.78 | -0.30 | 5.34E-04 | *Candida_24h* | Aspergillus_specific |
| rs1985993 | *ARF6* | 1033.95 | 0.38 | 2.04E-05 | *Candida_24h* | Aspergillus_specific |
| rs4685368 | *RFTN1* | 1418.71 | 0.50 | 5.60E-12 | *Candida_24h* | Aspergillus_specific |
| rs1985993 | *RPL36AL* | 1427.52 | 0.20 | 5.73E-03 | *Aspergillus_4h* | Aspergillus_specific |
| rs1985993 | *RPL36AL* | 1464.87 | 0.35 | 4.76E-02 | *Candida_4h* | Aspergillus_specific |
| rs1985993 | *ARF6* | 1485.71 | 0.30 | 2.41E-05 | *Aspergillus_4h* | Aspergillus_specific |
| rs1985993 | *RPS29* | 2172.08 | -0.34 | 3.93E-03 | *Candida_24h* | Aspergillus_specific |
| rs4685368 | *RFTN1* | 2583.93 | 0.27 | 2.50E-03 | *Candida_4h* | Aspergillus_specific |

Supplementary Table 3. ROS QTL loci influence the levels of cytokines in response to *C. albicans* in PBMCs. Cytokine QTL *P* values were derived after correcting for age, sex and cell count levels^9^.

| Chr | Pos | SNP/ROS QTL | Cytokine | Stimulus | beta | P value | eff | alt |
| --- | --- | --- | --- | --- | --- | --- | --- | --- |
| 2 | 239228138 | rs7571372 | IL22 | *C. albicans* yeast | 0.36 | 1.25E-03 | T | G |
| 2 | 239228138 | rs7571372 | IL22 | *C. albicans* hyphae | 0.23 | 4.94E-02 | T | G |
| 2 | 221804970 | rs34951328 | IL6 | *C. albicans* hyphae | 0.18 | 1.18E-02 | T | G |
| 9 | 136338580 | rs41297217 | TNFα | *C. albicans* hyphae | 0.32 | 4.03E-02 | G | A |
| 9 | 136338580 | rs41297217 | IL17 | *C. albicans* yeast | -0.31 | 4.15E-02 | G | A |
| 10 | 130403853 | rs12781072 | IL22 | *C.albicans* yeast | -0.30 | 3.64E-02 | T | A |
| 10 | 130403853 | rs12781072 | IL17 | *C.albicans* yeast | -0.25 | 3.68E-02 | T | A |
| 11 | 93012957 | rs10444213 | TNFα | *C. albicans* hyphae | -0.27 | 2.61E-03 | T | A |
| 10 | 1988696 | rs78346281 | TNFα | *A. famigatus conidia* | 0.11 | 4.66E-02 | C | T |

Abbreviations: Chr, chromosome;Pos, chromosomal position in base-pairs; eff, effective allele that tested for cytokine QTL effect; alt, alt, alternative allele

Supplementary Table 4. ROS-QTLs (P < 9.99 x 10^-6^) showed no significant association with candidaemia susceptibility. Summary statistics of GWAS candidaemia was previously described^11^.

|  |  |  |  |  |  | ROS-QTL mapping | | GWAS candidaemia | | | |  | |
| --- | --- | --- | --- | --- | --- | --- | --- | --- | --- | --- | --- | --- | --- |
| Chr | Pos | ROS QTL | Minor allele | Major allele | Fungus | P value | beta | F_A | F_U | P | OR | |  |
| 2 | 216300482 | rs1250259 | T | A | *A. fumigatus* | 8.43E-06 | 0.7 | 0.23 | 0.28 | 0.20 | 0.78 | |  |
| 2 | 103581470 | rs11893823 | T | G | *C. albicans* | 4.13E-07 | -0.84 | 0.45 | 0.45 | 0.87 | 0.97 | |  |
| 2 | 239228138 | rs7571372 | G | T | *C. albicans* | 2.32E-06 | 0.73 | 0.36 | 0.34 | 0.68 | 1.08 | |  |
| 2 | 216300482 | rs1250259 | T | A | *C. albicans* | 4.83E-06 | 0.72 | 0.23 | 0.28 | 0.20 | 0.78 | |  |
| 2 | 221804970 | rs34951328 | G | T | *C. albicans* | 6.06E-06 | -0.87 | 0.24 | 0.24 | 1.00 | 0.99 | |  |
| 2 | 238506581 | rs13391122 | T | C | *C. albicans* | 6.77E-06 | -0.81 | 0.19 | 0.23 | 0.33 | 0.81 | |  |
| 3 | 16681999 | rs4685368 | A | G | *A. fumigatus* | 5.56E-06 | 0.85 | 0.18 | 0.19 | 0.76 | 0.93 | |  |
| 3 | 174864481 | rs1381136 | A | G | *A. fumigatus* | 8.88E-06 | -0.61 | 0.37 | 0.41 | 0.29 | 0.84 | |  |
| 5 | 174600507 | rs57788948 | C | T | *A. fumigatus* | 3.63E-06 | 0.8 | 0.19 | 0.22 | 0.38 | 0.83 | |  |
| 9 | 136338580 | rs41297217 | A | G | *C. albicans* | 1.61E-06 | 1.33 | 0.08 | 0.10 | 0.48 | 0.80 | |  |
| 10 | 1988696 | rs78346281 | T | C | *A. fumigatus* | 6.46E-06 | 0.72 | 0.35 | 0.36 | 0.87 | 0.97 | |  |
| 10 | 127969267 | rs3858318 | G | A | *A. fumigatus* | 9.86E-06 | -0.96 | 0.11 | 0.12 | 0.71 | 0.91 | |  |
| 10 | 130403853 | rs12781072 | A | T | *C. albicans* | 6.00E-07 | 0.83 | 0.26 | 0.28 | 0.65 | 0.91 | |  |
| 11 | 93012957 | rs10444213 | T | A | *C. albicans* | 2.40E-06 | -0.73 | 0.45 | 0.47 | 0.58 | 0.91 | |  |
| 12 | 32074873 | rs10844056 | G | T | *A. fumigatus* | 4.90E-06 | -0.61 | 0.42 | 0.39 | 0.42 | 1.15 | |  |
| 12 | 32074398 | rs7342346 | G | A | *C. albicans* | 1.31E-06 | -0.65 | 0.42 | 0.39 | 0.42 | 1.15 | |  |
| 12 | 76417069 | rs6582326 | A | T | *C. albicans* | 8.21E-06 | -1.26 | 0.08 | 0.08 | 1.00 | 1.03 | |  |
| 14 | 50510387 | rs1985993 | A | G | *A. fumigatus* | 9.40E-06 | -1.18 | 0.11 | 0.09 | 0.42 | 1.25 | |  |
| 15 | 101807915 | rs2101171 | C | T | *A. fumigatus* | 7.39E-06 | 0.83 | 0.25 | 0.21 | 0.26 | 1.26 | |  |

Abbreviations: F_A, Frequency of this allele in cases; F_U, Frequency of this allele in controls
