# Supplementary figures and images for: Genetic determinants of fungi-induced ROS production are associated with the risk of invasive pulmonary aspergillosis
Source: Redox Biol. 2022 Jul 4;55:102391. doi: 10.1016/j.redox.2022.102391 (PMC9283926; doi:10.1016/j.redox.2022.102391)

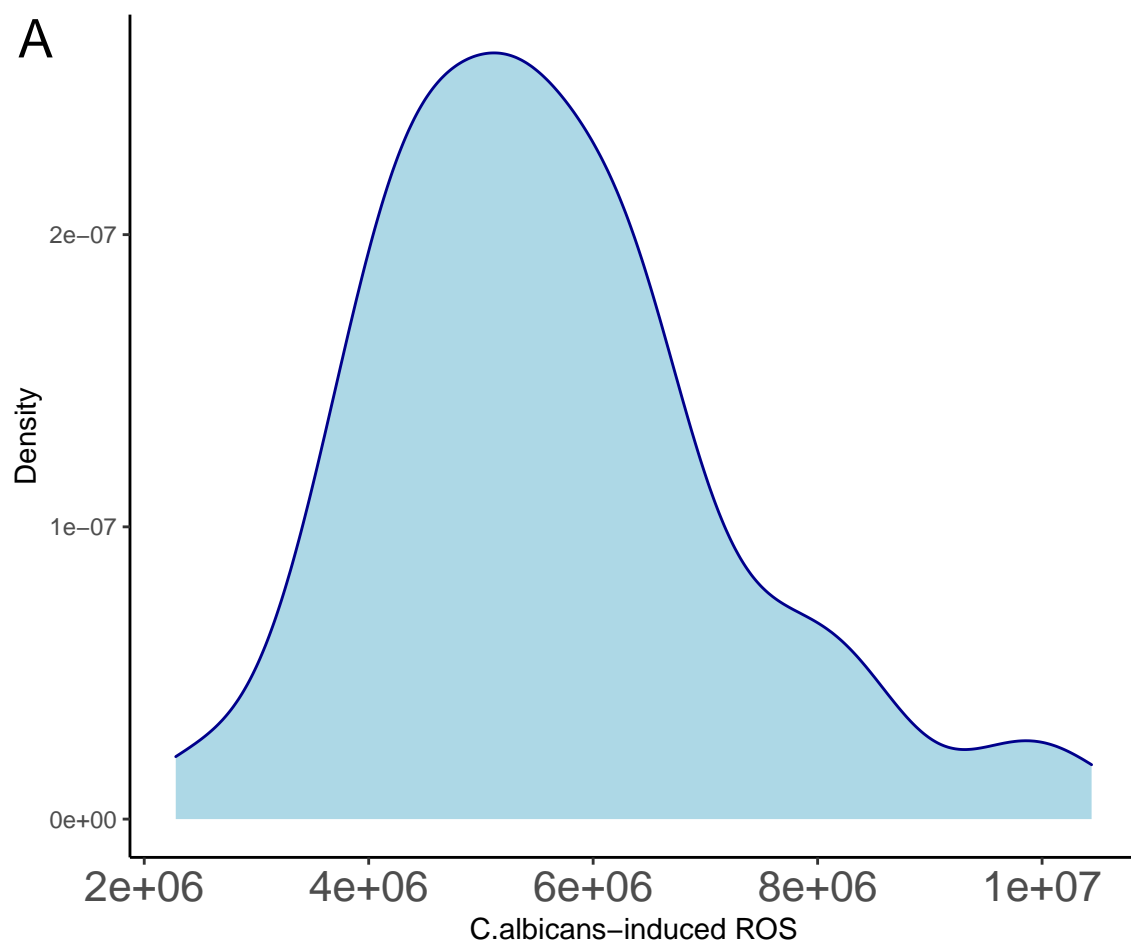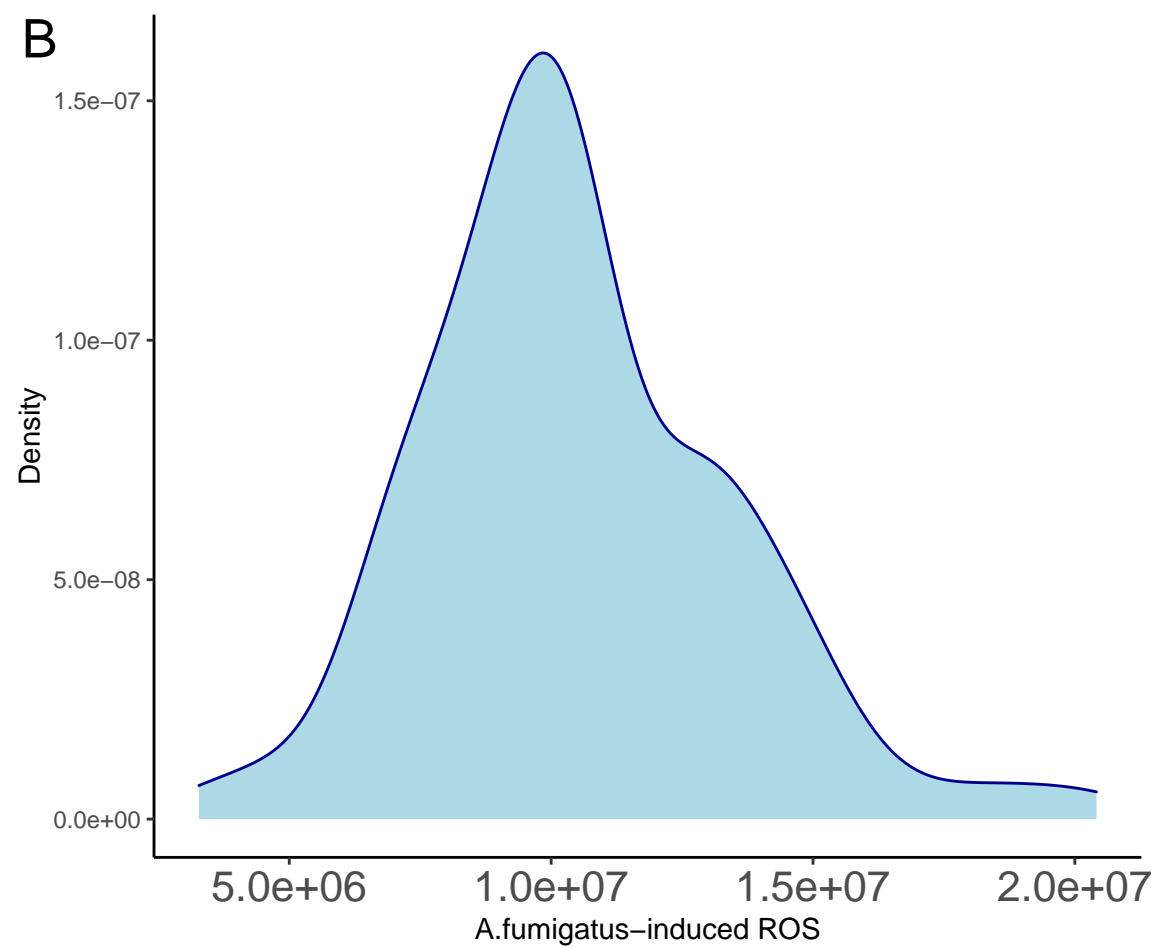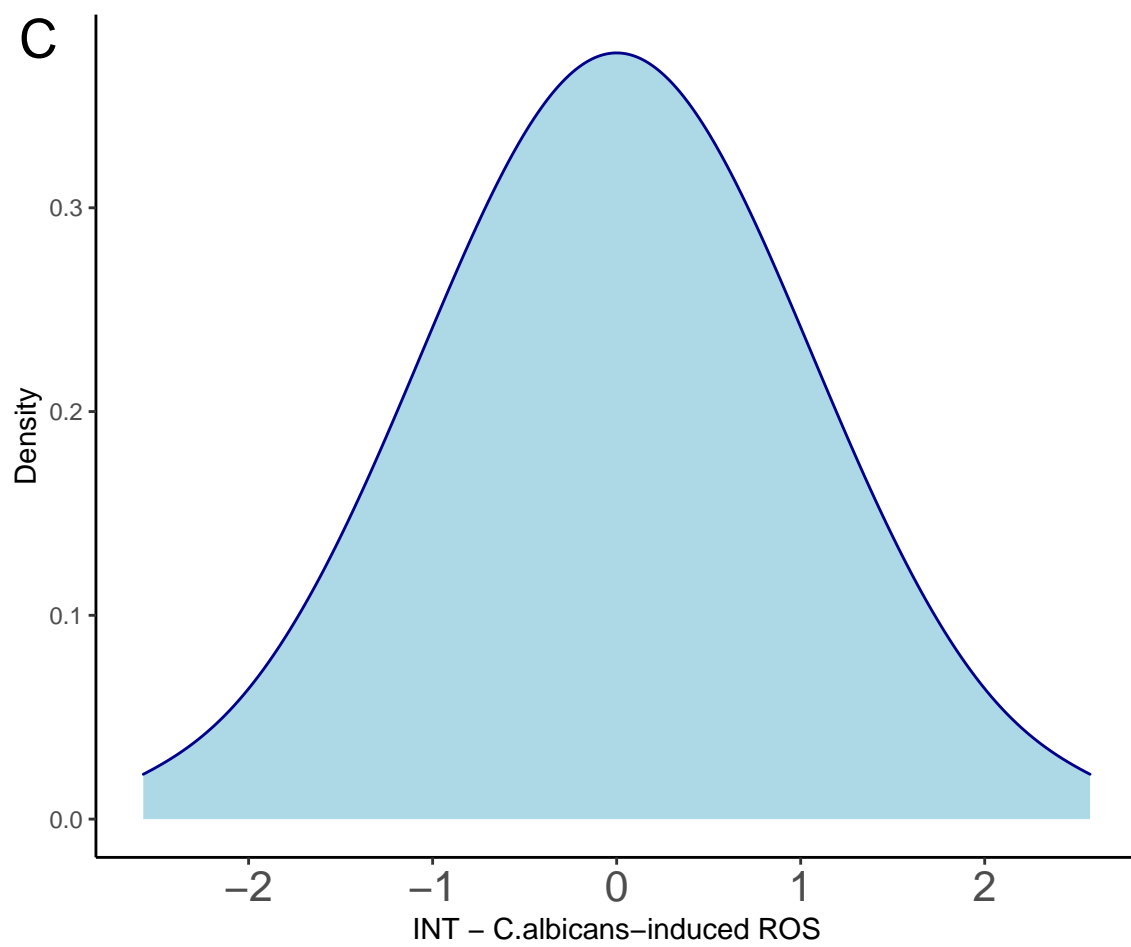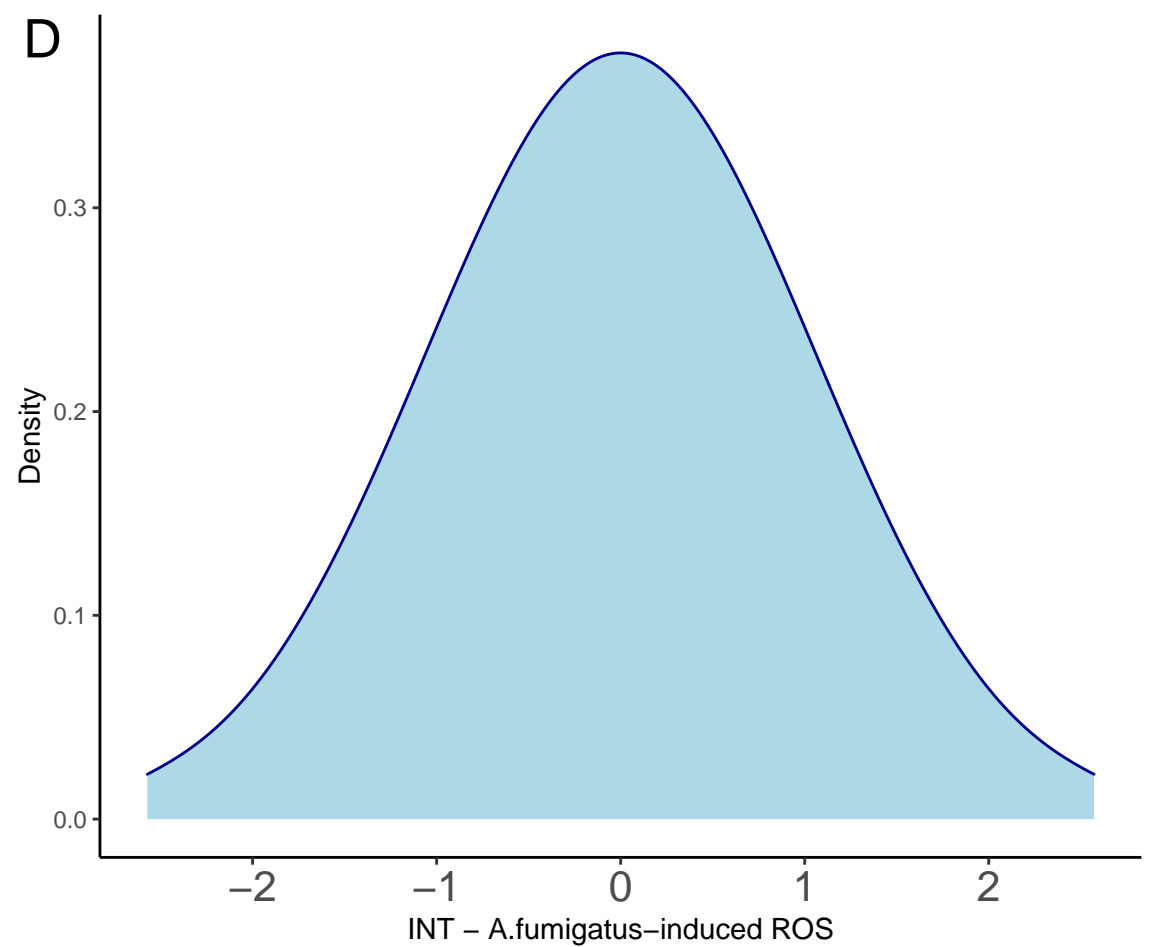

Supplement: Supplementary Fig. 1 — Distribution plots of (A and B) raw and (C and D) rank-based inverse transformed ROS levels in response to (A and C) C. albicans and (B and D) A. fumigatus. [file mmc2.pdf]

INT - *C. albicans* induced ROS

$R = 0.86, p < 2.2e-16$

2  
1  
0  
-1  
-2

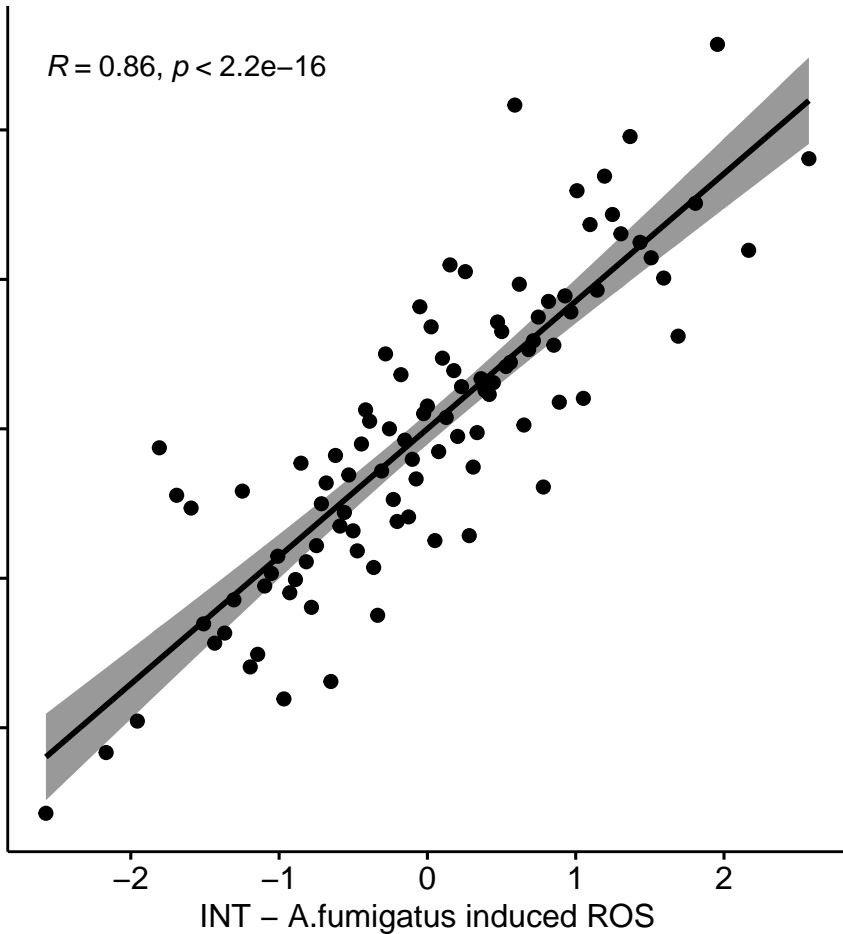

INT - *A. fumigatus* induced ROS

Supplement: Supplementary Fig. 2 — Correlation between ROS levels in response to C. albicans and A. fumigatus. Data were rank-based inverse transformed and correlation was calculated using Spearman's rank correlation. [file mmc3.pdf]
